# Robot‐assisted partial nephrectomy via retroperitoneal approach in a patient with horseshoe kidney

**DOI:** 10.1002/iju5.12297

**Published:** 2021-05-09

**Authors:** Shoko Uketa, Yousuke Shimizu, Ritsuki Yamaguchi, Noriaki Utsunomiya, Sojun Kanamaru

**Affiliations:** ^1^ Department of Urology Kobe City Nishi‐Kobe Medical Center Kobe Japan

**Keywords:** horseshoe kidney, renal cell carcinoma, robot‐assisted partial nephrectomy

## Abstract

**Introduction:**

Horseshoe kidney is a renal fusion anomaly often associated with ectopia, malformation, and vascular changes. Robot‐assisted partial nephrectomy is selected for patients with T1a renal cell carcinoma; however, there are few reports of renal cell carcinoma in horseshoe kidney. We present a case of robot‐assisted partial nephrectomy via a retroperitoneal approach in a patient with horseshoe kidney with a brief literature review.

**Case presentation:**

An 84‐year‐old woman presented with a 2‐cm mass in horseshoe kidney. She underwent robot‐assisted laparoscopic partial nephrectomy via a retroperitoneal approach.

**Conclusion:**

The use of robot‐assisted laparoscopic partial nephrectomy in patients with horseshoe kidney is very rare, and only four cases have been reported. Because of the unique anatomical structure, surgeons need to consider surgical strategy more carefully, considering tumor location, vascular anatomy, and past history of abdominal surgery.

**Abbreviations & Acronyms**
ccRCC: clear cell renal cell carcinoma
CT: computed tomography
HSK: Horseshoe Kidney
RAPN: robot‐assisted partial nephrectomy
RCC: renal cell carcinoma

**Keynote message**

We report a rare case of renal cell carcinoma in horseshoe kidney treated using robot‐assisted partial nephrectomy via a retroperitoneal approach.

## Introduction

RAPN has been covered by insurance for patients with RCC in Japan since 2016. Here, we performed RAPN in a patient with HSK; we report this rare case and review the literature.

## Case presentation

**Fig. 1:**
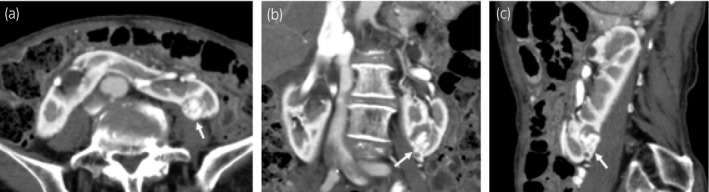
Computed tomography revealed a 2‐cm tumor (white arrow) in the left posterior aspect of the lower pole of the horseshoe kidney. (a) axial image; (b) coronal image; (c) sagittal image

**Fig. 2:**
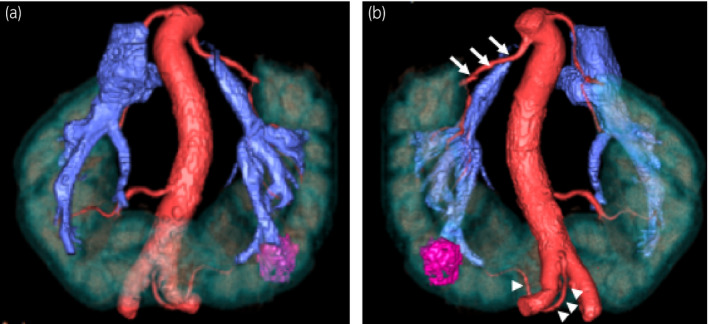
Three‐dimensional CT provided more detailed vessel information. There were two renal arteries and the tumor was located in a posterolateral region. One artery arose from the normal anatomic location (arrow) and the other arose from the dorsal side of the aortic bifurcation and snaked into the ventral side to nourish the tumor and isthmus (arrow head). (a) Anterior view. (b) Posterior view revealed the renal artery arising from the aortic bifurcation was also considered to be easily accessible via a retroperitoneal approach

An 84‐year‐old woman was hospitalized because a 2‐cm left kidney mass. It had been incidentally found on CT performed to determine the cause of vomiting (Fig. 1). In addition, she had a HSK. We examined the tumor location and vessel information in detail using three‐dimensional CT. The tumor was located in the posterolateral region of the lower pole. There were two renal arteries. One artery arose from the normal anatomic location and the other arose from the dorsal side of the aortic bifurcation and snaked into the ventral side to nourish the tumor and isthmus (Fig. 2).

**Fig. 3:**
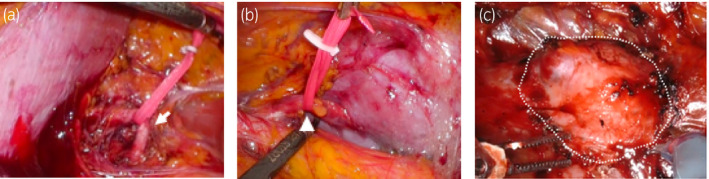
Intraoperative image. (a) One artery arose from the normal anatomic location (arrow). (b) The other arose from the dorsal side of the aortic bifurcation(arrow head). (c) The tumor was surrounded by white dotted line

Taking into consideration the abovementioned factors, we thought that it was easier to approach the tumor and arteries using a retroperitoneal approach. We started the operation in a conventional laparoscopic procedure. We cut the lateroconal fascia and could easily find the two renal arteries and secure them with vascular tape (Fig. 3a,b). We then switched to a robot‐assisted approach (da Vinci Xi). Because of the small amount of perirenal fat, we could find the tumor easily (Fig. 3c). The tumor was confirmed on ultrasonography, the two arteries were clamped, and the tumor was isolated with a sufficient margin. After excision of the tumor, bleeding from the resected margin was coagulated using a bipolar arm. The opening urinary tract was sutured using 3‐0 V‐Loc and the resected renal parenchyma was sutured using 2‐0 V‐Loc. We released the artery clamping and confirmed there was no persistent bleeding. Total operative time was 185 min and warm ischemia time was 18 min. The estimated blood loss was minimal. The pathological examination revealed clear cell RCC pT1a with negative surgical margins. She was discharged 8 days after surgery, and her clinical course was uneventful.

## Discussion

HSK is a renal fusion anomaly often associated with ectopia, malformation, and vascular changes found in about 0.15% to 0.25% of the population.^1^ Unique anatomic considerations, such as its low level of fixation, atypical and multiple vasculature changes, and the anterior renal pelvis, must be considered more carefully when contemplating surgery.^2^

Preoperative contrast‐enhanced imaging, such as CT or magnetic resonance imaging angiograms, should be perform to aid in delineating the vascular pattern as this is crucial when performing surgery in patients with HSK.

Partial nephrectomy as a surgical treatment for small‐diameter RCC instead of radical nephrectomy has become widespread.^3^ In addition, RAPN is more popular instead of open or laparoscopic partial nephrectomy due to its good visibility with 3D images and free operability of forceps^4^, ^5^; however, there are few reports of RAPN in patients with HSK.

To the best of our knowledge, there are five reported cases^6^, ^7^, ^8^, ^9^, ^10^ of conventional laparoscopic partial nephrectomy. Moreover, only four cases of RAPN in patients with HSK have been described in the literature (Table 1).^11^, ^12^, ^13^, ^14^ Due to its good visibility with 3D images and free operability of forceps, RAPN has the advantage of resection of the tumor and sutring of renal parenchyma compared with conventional laparoscopic procedures. However, the HSK requires extensive dissection to handle its poor mobility and identification of various vasculatures. Which is often difficult with RAPN because of a limited movement of robotic arm and requires conventional laparoscopic techniques.

**Table 1:** Summary of cases of RAPN in horseshoe kidney

Author	Age	Sex	Side	Size(cm)	Location	Number of renal artery	Operating time(min)	Console time(min)	WIT time(min)	Blood loss(mL)	Approach	Pathology
Raman et al^11^	53	Female	Right	3	Lower pole	2	170	120	13	150	Transperitoneal	Oncocytoma
Yamamichi et al^12^	62	Male	Left	1.6	Anterior middle pole	1	339	93	36	90	Retroperitoneal	ccRCC
Fujihara et al^13^	66	Male	Left	2	Posterior middle pole	3	Unknown	295	13	100	Transperitoneal	ccRCC
Numakura et al^14^	66	Male	Right	2.6	Posterior lower pole	2	290	Unknown	12	Unknown	Transperitoneal	ccRCC
Present case	84	Female	Left	2	Posterior lower pole	2	185	42	18	Minimal	Retroperitoneal	ccRCC

Three cases were treated via a transperitoneal approach and the other two, including our case, were treated via a retroperitoneal approach. Molina and Gill^6^ reported that because the unique anatomical structure can limit kidney mobilization, a different approach is recommended depending on the location of the renal mass. Tsivian et al.^7^ reported that surgeons should choose a transperitoneal approach if the tumor is located anteriorly, anterolaterally or in the isthmus, whereas a retroperitoneal approach is more suitable for posterior and posterolateral tumors.

We chose a retroperitoneal approach because of the tumor location and renal artery information. The tumor was located in a posterolateral region, and the renal artery arising from the aortic bifurcation was also considered to be easily accessible via a retroperitoneal approach (Fig. 2b).

However, Yamamichi, Fujihara, and Numakura's cases decided their approach for RAPN opposing this advice. In Yamamichi's case, the tumor was on the anterior side of the middle pole; however, the patient had a history of multiple abdominal surgeries. Therefore, a retroperitoneal approach was chosen.^12^ In Fujihara's case, the tumor was located posterolaterally and seemingly suitable for treatment using the retroperitoneal approach; however, they thought the transperitoneal approach would be better for performing the operation more easily because of the wider workspace and easier clamping of the artery.^13^

The tumor of Numakura's case was located in posterior, but they chose transperitoneal approach because the main arteries were running anteriorly. In addition, they combined laparoscopic procedure, which port placement was lower than the standard RAPN to allow for enough dissection.^14^

From the above, the most important factor is to choose an operative approach, and that decision depends on the patient's individual factors including tumor location, vascular anatomy, and history of abdominal surgery.

We made all possible assumptions during the preparation for the operation; for example, we considered dividing the isthmus using an Endo‐GIA stapler if the kidney was not sufficiently mobile. However, we were able to resect the tumor using minimal methods.

The age of robotic surgery is approaching and it is becoming possible to manage challenging cases that were difficult to treat with laparoscopic surgery. Surgeons should discuss preoperatively with precise preoperative imaging and more detailed information.

## Conclusion

We treated a rare case of RAPN in a patient with HSK. Surgeons need to plan a surgical strategy preoperatively considering the tumor location, vascular anatomy, and history of abdominal surgery.

## Ethics

We obtained written informed consent from the patient.

## Conflict of interest

The authors declare no conflict of interest.
